# Fluorescent tumour imaging of type I IGF receptor *in vivo*: comparison of antibody-conjugated quantum dots and small-molecule fluorophore

**DOI:** 10.1038/sj.bjc.6605103

**Published:** 2009-06-02

**Authors:** H Zhang, X Zeng, Q Li, M Gaillard-Kelly, C R Wagner, D Yee

**Affiliations:** 1 Masonic Cancer Center, University of Minnesota, Minneapolis, MN 55455, USA; 2 Department of Pharmacology, University of Minnesota, Minneapolis, MN 55455, USA; 3 Department of Chemistry, University of Minnesota, Minneapolis, MN 55455, USA; 4 Sanofi-aventis, Paris, France; 5 Department of Medicinal Chemistry, University of Minnesota, Minneapolis, MN 55455, USA

**Keywords:** type I IGF receptor, antibody, quantum dots, small-molecule fluorophore, tumour imaging

## Abstract

**Background:**

The type I insulin-like growth factor receptor (IGF1R) is a transmembrane tyrosine kinase involved in cancer proliferation, survival, and metastasis.

**Methods:**

In this study, we used two different fluorescent technologies (small-molecule fluorophores and quantum dot (QD) nanoparticles) to detect receptor expression and its downregulation by antibodies *in vivo*.

**Results:**

After conjugation with AVE-1642, a humanised anti-IGF1R monoclonal antibody, both QDs (705 nm) or Alexa 680 (small-molecule fluorophore) detected expression and downregulation of IGF1R *in vitro*. To examine their utility *in vivo*, either AVE-1642 conjugates were intravenously delivered to mice bearing xenograft tumours of mouse embryo fibroblasts expressing human IGF1R or MCF-7 human breast cancer cells. Quantum dot fluorescence was mainly localised to the reticuloendothelial system in several organs and engulfed by macrophages, with only very small amount of QDs detected in the xenograft tumours. Depletion of macrophages by clodronate liposomes did not alter the nonspecific uptake of QDs. In contrast, AVE-1642-conjugated Alexa 680 solely targeted to xenograft tumour and was able to detect IGF1R downregulation, with little nonspecific targeting to other tissues or organs in mice.

**Conclusion:**

Taken together, our data suggest that small-molecule fluorophores, not QDs, are suitable to detect the expression and downregulation of IGF1R *in vivo*.

In recent years, targeted therapies against specific membrane proteins have been developed as cancer treatments. One critical question in the development of this new class of drugs is to determine the expression of the target *in vivo.* Therapeutic benefit may be linked to the expression level of the molecular targets in the primary tumour site. For example, trastuzumab, the anti-HER2 antibody, is most effective in tumours overexpressing HER-2 (Nahta and Esteva, 2003). Therefore, the accurate assessment of HER-2 expression levels is essential for HER-2-targeted therapy. Certainly, the presence of the target is a necessary requirement for response to this type of drug.

The type I insulin-like growth factor receptor (IGF1R) is a receptor tyrosine kinase that plays critical roles in cancer progression and metastasis. Overexpression and activation of IGF1R has been reported in many types of cancer (Zhang and Yee, 2004, 2006). In the past few years, monoclonal antibodies and tyrosine kinase inhibitors have been developed to target IGF1R (Sachdev and Yee, 2006). Several anti-IGF1R monoclonal antibodies are in phase I, II, and III clinical trials. One interesting common feature about the antibodies is their ability to bind and downregulate IGF1R level through receptor-mediated endocytosis. Downregulation of IGF1R was associated with decreased tumour growth in xenograft tumour models (Burtrum *et al*, 2003; Maloney *et al*, 2003; Goetsch *et al*, 2005). Therefore, IGF1R downregulation could be used as a biodynamic marker of antibody delivery and a potential indicator of response.

In current anti-IGF1R clinical trials, patient enrolment is not based on the expression of IGF1R in the primary tumour. Although IGF1R is necessary for response to anti-IGF1R therapies, it is uncertain whether there is a relationship between patient response and levels of IGF1R expression in the tumour. Preclinical studies from Sanofi-aventis (Paris, France) have shown that there is no direct correlation between the antiproliferative effect of a human anti-IGF1R antibody, AVE-1642, and the level of IGF1R in more than 90 tumour cell lines (data not shown). Unlike HER-2, where expression levels are routinely measured by fluorescent *in situ* hybridisation or immunohistochemistry in clinical settings, a technique to quantitatively measure IGF1R level in tumour specimens has not yet been subjected to rigorous study. Moreover, there have not been reliable ways to measure receptor expression level *in vivo*. Recently, our laboratory has shown that anti-IGF1R antibody, which is specific for human IGF1R, when conjugated with quantum dots (QDs), has the ability to measure IGF1R level quantitatively (Zhang *et al*, 2008a).

Quantum dots are nanocrystals that emit fluorescence upon excitation. Compared with other types of fluorophore, QDs have high brightness and photostability (Zhang *et al*, 2008b). The recently developed cadmium telluride (Cd/Te) QDs emit fluorescence in the red and near-infrared range, which is ideal for *in vivo* imaging to avoid tissue auto-fluorescence. In fact, it has been applied to map sentinel lymph nodes in animal cancer models (Kim *et al*, 2004; Parungo *et al*, 2005; Soltesz *et al*, 2005, 2006; Hama *et al*, 2007; Knapp *et al*, 2007).

Although we have shown that AVE-1642-conjugated QDs are excellent agents to measure IGF1R level in cell lines with high specificity, their *in vivo* properties had not been investigated. As breast cancer metastasises to distant organs, it is often not clinically feasible to biopsy these tissues and measure levels of IGF1R in those sites. There is little evidence of gene amplification of IGF1R in breast cancer (Berns *et al*, 1992), and the level of IGF1R could vary from the primary tumour to metastatic tumours. Therefore, it is necessary to develop non-invasive *in vivo* imaging technology to quantitatively measure IGF1R levels in metastasised tumours and to be able to track the pharmacodynamic activity of antibody therapy.

In this study, the IGF1R-specific antibody, AVE-1642, was conjugated to Cd/Te QDs (with a peak emission at 705 nm). As a direct comparison, a small-molecule fluorophore, Alexa 680, with the same peak emission at 705 nm, was covalently linked to AVE-1642. We show that both antibody-conjugated Alexa 680, and QDs, localised to xenograft tumours that express IGF1R. However, QD localisation to the tumour was nonspecific and independent of antibody conjugation. Moreover, QDs were nonspecifically located in liver sinusoids. In contrast, only Alexa 680 fluorescence in tumour was dependent on IGF1R expression. Our results suggest that small-molecule fluorophores, such as Alexa 680, are more suitable for *in vivo* tumour imaging to identify IGF1R expression and its downregulation.

## Materials and methods

### Reagents

All chemical reagents were purchased from Sigma (St Louis, MO, USA) unless otherwise indicated. Optimum cutting temperature (OCT) compound was purchased from Sakura (San Marcos, CA, USA). AVE-1642 was developed by Sanofi-aventis and anti-CD20 antibody was a gift from ImmunoGen (Cambridge, MA, USA). The rat anti-mouse MOMA-2 antibody was purchased from AbD Serotec (Raleigh, NC, USA). SlowFade Gold antifade reagent with DAPI, goat anti-mouse Alexa Fluor 488, Qdot antibody conjugation kit, and the SAIVI rapid antibody labelling kit were purchased from Invitrogen (Carlsbad, CA, USA).

### Cell lines and culture

R cells (mouse fibroblast cells with a homozygous disruption of IGF1R gene) and R-/IGF1R cells (cell line derived from R cells with re-introduced IGF1R gene) were cultured according to literature (Sachdev *et al*, 2006). R cells were a gift from Renato Baserga (Thomas Jefferson University, Philadelphia, PA, USA) (Steller *et al*, 1996; Sachdev *et al*, 2006). MCF-7 cells were cultured in Dulbecco's modified Eagle's medium with 10% foetal bovine serum.

### Conjugation of antibody with QD 705 nm

AVE-1642 was conjugated to Cd/Te QDs (emission at 705 nm) through a heterobifunctional cross-linker, succinimidyl-4-(*N*-maleimidomethyl) cyclohexane-1-carboxylate (SMCC). The NHS ester end of SMCC reacted with the amine groups on the Cd/Te QDs, and the maleimide end of SMCC coupled to the sulfhydryls on the DTT-reduced antibody according to the instructions of the manufacturer (Invitrogen). Conjugate concentration was determined by the absorbance measured at 550 nm and was calculated using the extinction coefficient (1 700 000 M^−1^ cm^−1^).

### Conjugation of antibody with Alexa 680

AVE-1642 was conjugated to Alexa 680 using the SAIVI rapid antibody labelling kit from Invitrogen. The conjugate was purified by size exclusion column and its concentration was determined by the absorbance measured at 679 nm and calculated using the extinction coefficient (180 000 M^−1^ cm^−1^).

### Size exclusion chromatography

Gel filtration samples were fractionated with a Superdex G200 size exclusion column (GE Healthcare, Pittsburgh, PA, USA), eluted with P500 buffer (0.5 M NaCl, 50 mM KH_2_PO_4_, 1 mM EDTA, pH 7.0), and the relative peak retention time quantified by absorbance at 280 nm. The Superdex G200 column was calibrated with a molecular weight standards kit (Sigma). The test reagents were blue dextran (MW=2000 kDa), thyroglobulin (669 kDa), apoferritin (443 kDa), *β*-amylase (200 kDa), alcohol dehydrogenase (150 kDa), bovine serum albumin (66 kDa), and carbonic anhydrase (29 kDa).

### Flow cytometry

Cells were trypsinised and resuspended in PBS/1% BSA/0.1% sodium azide (FACS buffer). Cells were incubated with free or conjugated QDs or Alexa 680 in FACS buffer for 1 h at 4 °C. Cells were washed twice and resuspended with 400 *μ*l FACS buffer. IGF1R levels on cell surface were measured using an LSRII flow cytometer (BD Biosciences, San Jose, CA, USA). Quantum dot 705 nm fluorescence was obtained with a band filter at 700/40 nm using an excitation laser at violet. Alexa 680 fluorescence was obtained with a band filter at 700/40 nm using an excitation laser at 633 nm.

### Tumour growth in athymic mice and delivery of antibody-conjugated fluorophores

All animal studies were performed under the guideline of a university-approved animal care and use protocol (protocol no. 0511A77590 and renewed protocol no. 0807A40961). Female athymic mice, 4–5 weeks old (Foxn1nu strain from Harlan Sprague Dawley, Indianapolis, IN, USA), were injected in the second or seventh mammary fat pad with 5 × 10^6^ R-/IGF1R cells or MCF-7 cells. For mice injected with MCF-7 cells, mice also were implanted with a slow-release capsule of 17-*β* oestradiol (Sigma) subcutaneously in the dorsal neck region 1 day before the cell injection. When tumour volume reached 100–300 mm^3^, approximately 0.1 nmol of antibody-conjugated Alexa 680 or QDs (at about 200 *μ*l volume) was injected intravenously into the tail vein of mice. For antibody pretreatment experiment, 200 *μ*g of AVE-1642 was injected into mice intraperitoneally.

### Clodronate liposome preparation and delivery into mice

Clodronate liposome or the PBS liposome was prepared according to literature (Van Rooijen and Sanders, 1994). About 200 *μ*l of clodronate liposome or PBS liposome was administered into mice through tail vein injection.

### *In vivo* animal imaging

Right before imaging, mice were anaesthetised in a closed chamber with isoflurane gas (administered in conjunction with pure oxygen). Then mice were quickly translocated into the imaging chamber of the Maestro *in vivo* fluorescence imaging system (CRI, Woburn, MA, USA). The heads of mice were inserted into the nose cone with continuous isoflurane gas flow to keep them anaesthetised during the imaging process. Fluorescence was excited with an excitation filter at 575–605 nm and images were captured at the 645–850 nm range in 10 nm steps with an emission filter 645LP. Raw, mixed signal images were analysed by the Maestro 2.2 software to isolate the autofluorescence (based on the control animal) and QD (or Alexa 680) fluorescence.

### Tumour *ex vivo* imaging

Xenograft tumours were removed from the surrounding tissue soon after mice were killed by CO_2_ overdose. Tumours were cut open and placed in the imaging chamber, and tumour fluorescence was captured by the Maestro *in vivo* imaging system and analysed by the Maestro 2.2 software.

### Liver sample preparation

Liver specimens were taken right after mice were killed by CO_2_ overdose and frozen using the OCT compound in liquid nitrogen. Liver samples were sectioned at 8 *μ*m thickness.

### Toluidine blue staining

After fixation with acetone, liver sections were placed in distilled water for 1 min and then into a 1% toluidine blue O solution, pH 5.5, for 1 min. Finally, slides were mounted with SlowFade Gold antifade reagent.

### Immunofluorescent staining

After blocking in casein block solution, liver sections were incubated with rabbit anti-mouse MOMA-2 antibody (1 : 25). The 4 °C overnight primary incubation was followed by secondary applications with goat anti-mouse Alexa Fluor 488 (1 : 250). Finally, slides were mounted with SlowFade Gold antifade reagent with DAPI.

### Confocal microscopy

Confocal laser scanning microscopy was performed with an Olympus Fluoview FV500 laser scanning confocal system (Olympus, Center Valley, PA, USA), using a × 60 oil immersion objective. Excitation lasers and filters were as follows: DAPI, blue diode laser, emission 430–460 nm; MOMA-2, argon laser, emission 505–525 nm; Alexa 680, red He–Ne laser, emission LP 660 nm; and QD 705 nm, blue diode laser, emission LP 660 nm.

## Results

### Molecular weights of QDs and Alexa 680 conjugates measured by size exclusion chromatography

**Figure 1:**
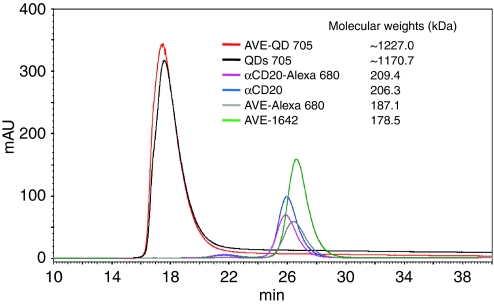
QD conjugates had a much larger molecular weight compared with Alexa 680 conjugated. Gel filtration samples, including AVE-1642 QDs (AVE-QD 705), QDs, anti-CD20 Alexa 680 (*α*CD20-Alexa 680), anti-CD20 antibody, AVE-1642 Alexa 680 (AVE-Alexa 680), and AVE-1642, were fractionated with a Superdex G200 size exclusion column, eluted with P500 buffer (0.5 M NaCl, 50 mM KH_2_PO_4_, 1 mM EDTA, pH 7.0) and the relative peak retention time quantitated by absorbance at 280 nm. Molecular weights of the conjugates were calculated according to a molecular weight standards kit.

The IGF1R-specific antibody, AVE-1642, was conjugated to the Cd/Te QDs and Alexa 680, respectively. A control anti-CD20 antibody, which binds only CD20 protein expressed on the surface of mature B cells, was conjugated to Alexa 680. Size exclusion chromatography was performed to measure the molecular weights of the conjugates. As shown in Figure 1, pure QDs and AVE-1642 QDs have calculated molecular weights of 1170.7 and 1227.0 kDa, respectively, much larger than those of the Alexa 680 conjugates. As Alexa 680 is a small molecule, its conjugation to antibodies did not have a significant effect on molecular mass compared with antibody alone.

### Specific binding of AVE-1642-conjugated QDs and Alexa 680 to cells that express IGF1R *in vitro*

**Figure 2:**
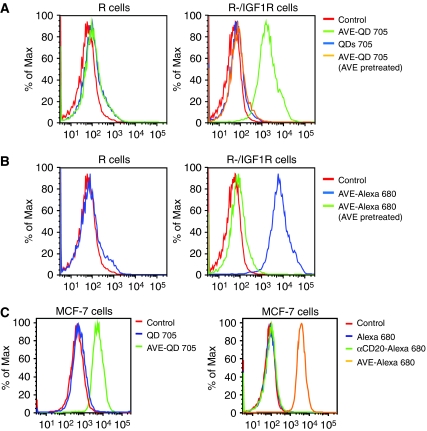
Both AVE-1642-conjugated QDs and Alexa 680 bound to cells that express IGF1R. R cells, R-/IGF1R cells, or MCF-7 cells were trypsinised, resuspended in FACS buffer, and incubated with pure QDs, AVE-1642-conjugated QDs (AVE-QD 705) (**A**), AVE-1642-conjugated Alexa 680 (AVE-Alexa 680) (**B**), or both types of conjugates, including an anti-CD20 Alexa 680 conjugate (**C**) for 1 h. In addition, R-/IGF1R cells were pretreated with AVE-1642 (AVE) antibody for 24 h, and then incubated with AVE-QDs or AVE-Alexa 680. The fluorescence of bound QDs, or Alexa 680, was analysed by flow cytometry. The horizontal axis of the diagram represents the fluorescent intensity, and the vertical axis shows the percentage of maximum cell number.

Pure unconjugated QDs, or AVE-1642-conjugated QDs, were incubated with R cells, a mouse embryo fibroblast cell line that has genetic deletion of the IGF1R gene. After washing with FACS buffer, bound cell fluorescence was analysed by flow cytometry. No specific fluorescence was detected on cell surface. When R-/IGF1R cells, a cell line stably transfected with a human *igf1r* cDNA, were incubated with pure QDs or AVE-1642 QDs, only AVE-1642 QDs showed bound fluorescence. Pure QDs failed to bind to cell surface. In addition, if R-/IGF1R cells were pretreated with AVE-1642 antibody to downregulate IGF1R level, AVE-1642 QDs no longer bound to cell surface with a diminished IGF1R level (Figure 2A). Similar results were obtained with the AVE-1642-conjugated Alexa 680 (Figure 2B).

The ability of AVE-1642-conjugated QDs and Alexa 680 to bind to MCF-7 cells, a breast cancer cell line that expresses high level of IGF1R, was also examined. As shown in Figure 2C, AVE-1642 QDs, but not pure QDs, bound to MCF-7 cells. AVE-1642-conjugated Alexa 680, but not Alexa 680 alone, or the anti-CD20 conjugates, bound to MCF-7 cells. In addition, we confirmed that the anti-CD20 Alexa 680 conjugates recognised B cells specifically by flow cytometry (data not shown).

Therefore, our results suggest that *in vitro,* both AVE-1642-conjugated QDs and Alexa 680 were able to detect IGF1R expression and its downregulation with similar affinity.

### Uptake of QD fluorescence in mice carrying xenograft tumours

Athymic mice, 4–5 weeks old, were injected with R-/IGF1R cells to form xenograft tumours. When tumour volumes reach 100–300 mm^3^, equal amounts of pure QDs, or AVE-1642 QDs (0.1 nmol), were injected into the tail vein of the mice. Blood was drawn and the intensity of QD fluorescence was examined using the Maestro *in vivo* imaging system. Quantum dot fluorescence diminished very rapidly, and by 2 h, QD fluorescence in circulation was undetectable by fluorescence imaging (data not shown). Therefore, whole-body *in vivo* imaging was performed at 2 and 24 h after QD injection.

**Figure 3:**
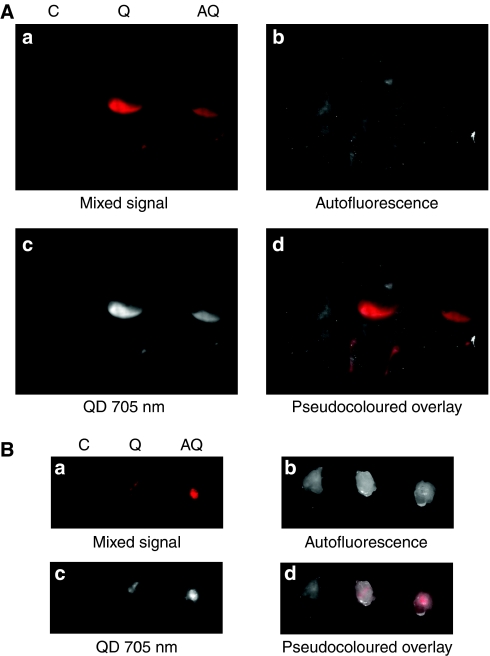
QD fluorescence was mainly localised to the liver, spleen, lymph nodes, and bone marrow, with small amount of tumour targeting. (**A**) Mice carrying R-/IGF1R xenograft tumours in the left (no. 2) mammary fat pad were injected with PBS solution (C), 0.1 nmol of QDs (Q), or AVE-1642 QDs (AQ) through tail vein. Mice were imaged after 2 h using the Maestro *in vivo* imaging system. (**a**) Mixed raw signal captured by Maestro. (**b**) Autofluorescence after spectral unmixing. (**c**) QD 705 nm fluorescence after spectral unmixing. (**d**) Pseudocoloured overlay, with autofluorescence as white colour and QD fluorescence as red colour. (**B**) Mice were killed and tumours were removed. Tumour *ex vivo* imaging was performed using the Maestro system.

With an excitation source at 575–605 nm, Maestro *in vivo* imaging system was used to capture fluorescence at 10 nm stepwise from 645 to 850 nm. After mixed raw images were captured, Maestro 2.2 software was used to perform spectral unmixing and to isolate autofluorescence and QD fluorescence. Finally, autofluorescence image and QD fluorescence image were overlayed with pseudocolours to show the localisation of QD signal in the whole body. As shown in Figure 3A, by 2 h, there was a large amount of QD fluorescence in the liver, spleen, lymph nodes, and bone marrow. These are all organs that contain the reticuloendothelial system (RES), where the phagocytic cells are present in large quantity. In addition, the capillary beds of these organs are all highly permeable sinusoids, with a discontinuous endothelium.

The strong nonspecific uptake obscured any tumour uptake in the intact animal. To evaluate tumour-specific uptake, mice were killed and xenograft tumours were removed and subjected to *ex vivo* imaging. As shown in Figure 3B, tumours in mice injected with pure QDs and AVE-1642 QDs both had small amount of fluorescence, especially in the centre of the tumour. However, multiple experiments showed that there was not a substantial difference between the fluorescence of unconjugated QDs and AVE-1642 QDs located in tumours. In addition, downregulation of IGF1R levels by antibody pretreatment did not show a significant difference in AVE-QD fluorescence targeting (data not shown). Thus, QDs could localise to the tumour, but the localisation was not specific for IGF1R expression.

To exclude the possibility that the AVE-1642 chemical linkage with QDs was unstable in serum and resulted in the dissociation of the antibody with QDs, we developed an alternative linkage method to conjugate AVE-1642 with QDs. The free carboxyl groups of any aspartic and glutamic residues in the antibody were activated with EDC/sulpho-NHS, and then directly conjugated to the amine group on the QDs. Theoretically, the bond between AVE-1642 and QDs would be as stable as the peptide bond in AVE-1642. These ‘direct-linkage AVE-1642 QDs’ bound to IGF1R-expressing cells *in vitro*, and they did not affect the nonspecific uptake or tumour targeting *in vivo* (data not shown).

### QD cellular localisation in the liver

**Figure 4:**
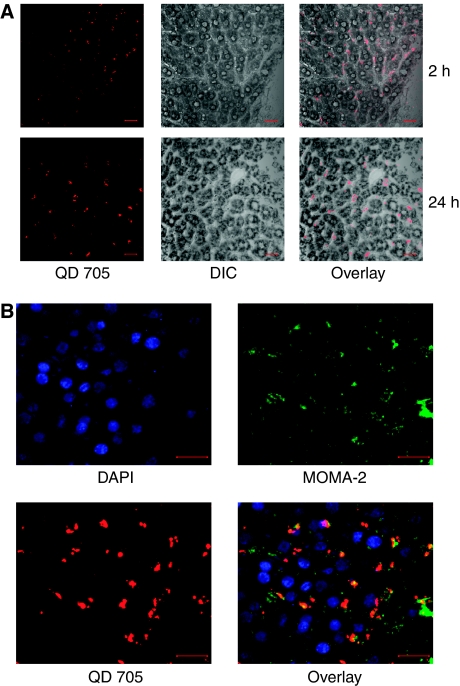
QDs were localised to the hepatic sinusoids and engulfed by Kupffer cells. (**A**) After AVE-1642 QDs were delivered systematically for 2 h (top panel) or 24 h (bottom panel), mice were killed and liver pieces were frozen in OCT solution. Liver sections were stained with toluidine blue and imaged by confocal microscopy. QD 705 fluorescence is shown in the left, DIC image is shown in the middle, and an overlay image is shown in the right. The scale bar in the image is 20 *μ*m. (**B**) Liver sections with AVE-1642 QDs delivered for 2 h were stained with anti-MOMA-2 antibody, followed by an Alexa 488-conjugated secondary antibody. Finally, the sections were stained for the nucleus with DAPI. Confocal microscopy was performed to examine the localisation of QD fluorescence in the liver tissue. The scale bar in the image is 20 *μ*m.

To detect the anatomic location of the QDs in the liver, liver sections were stained with toluidine blue to facilitate differential interference contrast (DIC) imaging. Overlay of QD fluorescence and the DIC imaging of the liver showed that AVE-1642 QDs were localised in the hepatic sinusoids at 2 and 24 h post-injection, despite the fact that the sinusoids are highly permeable (Figure 4A). A specific antibody against MOMA-2, a cell marker for monocytes and macrophages, showed that QD fluorescence co-localised with MOMA-2 staining (Figure 4B), suggesting that QDs were engulfed by Kupffer cells, the liver-specific macrophages. Same results were obtained for the pure QDs (data not shown).

### QD tumour targeting was not affected by macrophage depletion

**Figure 5:**
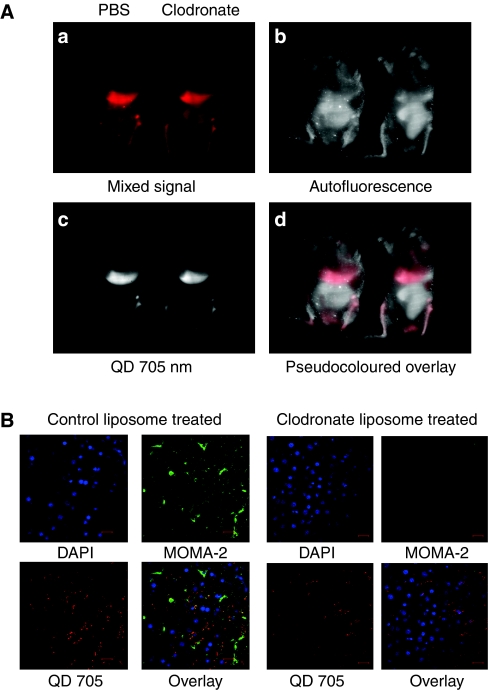
Depletion of macrophages failed to decrease nonspecific uptake. (**A**) Mice carrying R-/IGF1R xenograft tumours were administered with PBS or clodronate liposome. After 24 h, mice were tail vein injected with 0.1 nmol of AVE-1642 QDs. Two hours later, whole-body imaging was performed with the Maestro *in vivo* imaging system. (**a**) Mixed raw signal captured by Maestro. (**b**) Autofluorescence after spectral unmixing. (**c**) QD 705 nm fluorescence after spectral unmixing. (**d**) Pseudocoloured overlay with autofluorescence as white colour and QD fluorescence as red colour. (**B**) After whole-body imaging, mice were killed and liver sections were stained for MOMA-2. Confocal microscopy was performed to examine the presence or absence of MOMA-2.

It has been hypothesised that the phagocytosis by macrophages in the RES system is a major driving force for the nonspecific hepatic uptake (Simberg *et al*, 2007). To decrease the nonspecific uptake by organs with RES system, and increase the chance of QDs to reach tumour, we treated mice with clodronate liposomes to temporarily deplete macrophages in the RES system. Clodronate liposomes, once phagocytosed by macrophages, accumulate inside the cell and cause cell apoptosis (Van Rooijen and Sanders, 1994). However, macrophage depletion did not prevent the nonspecific uptake, and identical imaging patterns were obtained after AVE-1642 QDs were delivered (Figure 5A). After whole-body imaging, mice were killed and liver sections were stained with MOMA-2, the cell marker for macrophages. Confocal microscopy image confirmed that the majority of Kupffer cells had been successfully depleted by the clodronate liposome (Figure 5B). Thus, our data show that QDs undergo phagocytosis by Kupffer cells in liver sinusoids; however, QD localisation to the liver is independent of this process.

### Specific tumour targeting of AVE-1642 Alexa 680

**Figure 6:**
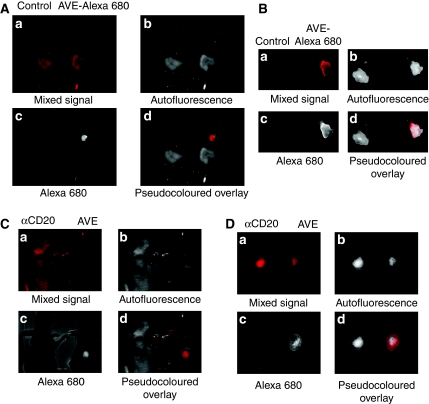
AVE-1642 Alexa 680 solely targeted to tumours that express IGF1R. (**A**) Mice carrying R-/IGF1R xenograft tumours were injected through tail vein with either PBS or 0.1 nmol of AVE-1642 Alexa 680 (AVE-Alexa 680). Imaging was taken 48 h later. (**a**) Mixed raw signal captured by Maestro. (**b**) Autofluorescence after spectral unmixing. (**c**) Alexa 680 fluorescence after spectral unmixing. (**d**) Pseudocoloured overlay with autofluorescence as white colour and Alexa 680 fluorescence as red colour. (**B**) After *in vivo* imaging, mice were killed. Tumour *ex vivo* images were taken using the Maestro system. (**C**) Mice carrying MCF-7 xenograft tumours were injected through tail vein with either anti-CD20 Alexa 680 (*α*CD20) or AVE-1642 Alexa 680 (AVE). Imaging was taken 48 h later. (**D**) After *in vivo* imaging, mice were killed. Tumour *ex vivo* images were taken using the Maestro system.

As a direct comparison, equal amounts of AVE-1642-conjugated Alexa 680 (0.1 nmol) were injected into mice carrying R-/IGF1R xenograft tumours. Whole-body imaging was performed at 2 h, 5 h, 1 day, 2 days, 4 days, and 10 days time frame. Starting at day 1, we observed specific Alexa 680 fluorescence in the xenograft tumours. Figure 6A shows images captured after 2 days of Alexa 680 administration. The fluorescence lasted for at least 4 days and diminished after 10 days. Uptake was not observed in any mouse organ or tissue (Figure 6A). In addition, tumour *ex vivo* imaging confirmed the Alexa 680 fluorescence at the tumour sites (Figure 6B). Imaging of the organs in the chest and abdominal region of the mice reveals no Alexa 680 fluorescence (data not shown).

To exclude the possibility that Alexa 680 by itself may target to tumours, equal amounts of AVE-1642-conjugated Alexa 680 or anti-CD20-conjugated Alexa 680 were injected into mice carrying MCF-7 xenograft tumours. Whole-body imaging was performed after 2 days. As shown in Figure 6C, only AVE-1642-conjugated Alexa 680 accumulated at the tumour. Anti-CD20 conjugates failed to accumulate specifically in mouse body. Tumour *ex vivo* imaging confirmed the *in vivo* results at the tumour sites (Figure 6D).

### Detection of IGF1R downregulation in xenograft tumours by AVE-1642 Alexa 680

**Figure 7:**
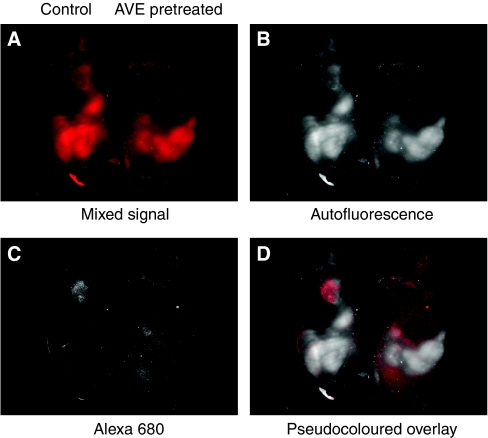
AVE-1642 Alexa 680 detected IGF1R downregulation. Mice carrying R-/IGF1R xenograft tumours were treated with or without 200 *μ*g of AVE-1642 antibody. Two days later, mice were injected through tail vein with 0.1 nmol of AVE-1642 Alexa 680. Mice whole-body imaging was taken 24 h later. (**A**) Mixed raw signal captured by Maestro. (**B**) Autofluorescence after spectral unmixing. (**C**) Alexa 680 fluorescence after spectral unmixing. (**D**) Pseudocoloured overlay with autofluorescence as white colour and Alexa 680 fluorescence as red colour.

Next, we studied whether AVE-1642-conjugated Alexa 680 targeting to tumours was dependent on IGF1R expression. Mice carrying R-/IGF1R tumours were pretreated with AVE-1642 antibody for 2 days before the injection of AVE-1642-conjugated Alexa 680, which efficiently downregulates IGF1R levels in tumours (Sachdev *et al*, 2006). As shown in Figure 7, the tumours with diminished IGF1R levels showed a dramatically decreased fluorescence, compared with the tumours with a regular amount of IGF1R level. Therefore, our data suggest that AVE-1642-conjugated Alexa 680 can identify IGF1R expression and its downregulation in tumour xenograft models.

## Discussion

Anatomic imaging is commonly used to gauge the efficacy of cancer therapy. However, current *in vivo* imaging techniques, including X-ray, computerised axial tomography, ultrasound, nuclear imaging, and magnetic resonance imaging, do not have the ability to detect specific cancer cell proteins that serve as molecular targets. Investigational studies utilising positron emission tomography and scintigraphy have shown that it is possible to detect HER2 and ER *in vivo* (Linden *et al*, 2006; Perik *et al*, 2006), and an increased need to visualise these tumour targets is apparent as targeted therapies against specific molecules in cancer are rapidly developing.

Given the critical role of IGF1R in cancer biology, many new anti-IGF1R drugs have been developed, including monoclonal antibodies and tyrosine kinase inhibitors. Several monoclonal antibodies have shown early promise in clinical trials. However, an *in vivo* imaging technique, to identify appropriate patients for anti-IGF1R therapy, and to track the delivery of such a therapy, is not available. Therefore, there is an important clinical need to develop *in vivo* imaging techniques to detect and measure the level of the molecules *in vivo* non-invasively. Owing to the high brightness of QDs, they have been studied extensively for their application in cancer imaging. However, through direct comparison, our data have shown that small-molecule fluorophores, conjugated with monoclonal antibodies, are more suitable to detect IGF1R-expressing tumours *in vivo* through non-invasive fluorescent imaging.

Our data have shown that both AVE-1642-conjugated QDs and Alexa 680 bind specifically to IGF1R-expressing cells. Furthermore, they both detect the downregulation of IGF1R after treatment with a monoclonal antibody. Direct conjugation of AVE-1642 with QDs or Alexa 680 does not alter the ability of AVE-1642 to bind IGF1R. These data suggest that both agents could be used clinically *in vitro* to examine the IGF1R level in primary tumours. As the fluorescence intensity correlates with IGF1R level, antibody-conjugated fluorophores can be used to quantitatively measure IGF1R levels by flow cytometry.

In the past few years, QDs, especially the Cd/Te QDs with emission wavelengths at near-infrared region, have been studied intensively for cancer imaging. Several groups have shown that after systematic administration, antibody- or ligand-conjugated QDs can localise to tumour (Gao *et al*, 2004; Cai *et al*, 2006; Tada *et al*, 2007; Diagaradjane *et al*, 2008). However, it was not clear how efficient conjugated QDs can reach and bind specific receptors on tumour cells after intravenous delivery *in vivo*. Recently, Cai *et al* (2006) have shown that RGD (Arg-Gly-Asp) peptide-conjugated QDs that specifically recognise *α*_v_*β*_3_ integrin localised to tumour with a heterogeneous pattern. At the microscopic level, they discovered that QDs were retained in the tumour vasculature and did not appear to contact the tumour cells (Cai *et al*, 2006). Diagaradjane *et al* (2008) also found that an EGF-conjugated QD bound to EGFR in tumour vasculature after systematic delivery. Consistent with their data, in our studies, both pure QDs and AVE-1642-conjugated QDs were localised to tumours with a heterogeneous pattern, suggesting that they localised to tumour vasculature. As AVE-1642 antibody specifically binds human IGF1R and does not bind to mouse IGF1R, the staining pattern we saw was not due to detection of murine IGF1R on endothelial cells.

It has been a consistent observation that systemic administration of pure, non-targeted QDs, and cell-targeted QDs, accumulates in substantial quantities in the liver, spleen, lymph nodes, and bone marrow in animal model systems (Ballou *et al*, 2004, 2007; Gao *et al*, 2004; Cai *et al*, 2006; Jackson *et al*, 2007; Diagaradjane *et al*, 2008; Li and Huang, 2008). These organs all contain the RES system, where macrophage cells reside. We have shown that QDs or conjugated QDs accumulated inside the sinusoids in the liver and were subsequently phagocytosed by Kupffer cells. Our data were consistent with others' findings that QDs in the liver tend to be engulfed by macrophages (Jackson *et al*, 2007). However, when we deliberately depleted macrophages by clodronate liposomes, we still observed that QDs accumulated in the RES-containing organs with similar kinetics. Therefore, macrophage engulfing was not the proximal cause of preferential localisation. What causes the localisation to RES-containing organs is unclear. It is noteworthy that virtually all nano-size crystals, including nano-sized liposomes (Li and Huang, 2008), QDs (Zhang *et al*, 2008b), and iron oxide nanocrystals (Simberg *et al*, 2007), tend to accumulate in these RES-containing organs. Therefore, it is likely that the size of these particles has a critical role in the nonspecific uptake. A recent study suggests that smaller QDs (with a size less than 5.5 nm), but not large QDs, could be cleared through renal excretion (Choi *et al*, 2007). However, small-size QDs normally have short emission wavelengths, which are not suitable for *in vivo* imaging. Quantum dots with longer emission wavelengths may be retained in the body for at least 2 years and remain fluorescent (Ballou *et al*, 2007). This nonspecific uptake will certainly hinder the application of QDs in clinical settings. Even if the tumour uptake was specific, the long-term consequences of QDs in the liver would need to be understood before clinical applications could proceed.

Beyond this concern about the nonspecific uptake in the RES-containing organs, our data show that QD-conjugated antibodies were unable to specifically localise to tumours. Thus, this approach would not be suitable for *in vivo* imaging to target molecules on tumour surface. Although QD-conjugated antibodies performed well *in vitro*, there are certainly less complicated ways to measure cell-surface receptor expression in tumours removed from the body.

In contrast, small-molecule fluorophores, such as Alexa 680, coupled with AVE-1642 had the ability to identify IGF1R expression *in vivo*. As the size of Alexa 680 is much smaller than that of the antibody, it seemed that the pharmacodynamics of AVE-1642 conjugated to Alexa 680 is similar to that of the antibody alone. Without nonspecific uptake in the RES-containing organs, AVE-1642 Alexa 680 circulated long enough to accumulate in the xenograft tumour. In addition, previous studies in our lab have shown that AVE-1642 targeted to tumour and downregulate IGF1R levels in tumour after intraperitoneal injection. AVE-1642 Alexa 680 could monitor this downregulation of IGF1R by diminished tumour fluorescence. As downregulation of IGF1R was associated with decreased tumour growth (Burtrum *et al*, 2003; Maloney *et al*, 2003; Goetsch *et al*, 2005), AVE-1642 Alexa 680 could be used to predict response post-therapy by fluorescent imaging. We also realise that our study utilised optimal conditions as AVE-1642 does not bind mouse IGF1R; therefore, the potential background generated by mouse IGF1R was avoided. It is important in future studies to include anti-IGF1R antibodies, such as A12 (Wu *et al*, 2005; Rowinsky *et al*, 2007), which recognise both human and mouse IGF1R, to examine the tumour-to-background signal ratio from regions of interest in tumour and normal tissue.

In summary, our data, coupled with others' finding, suggest that imaging particles at nano-size tend to have nonspecific uptake, either alone or conjugated with antibody. In contrast, small-molecule probes conjugated to antibody are more specific and are able to reach tumour sites. On the basis of these characteristics, we anticipate that antibody-conjugated small-molecule probes will be able to localise to metastasised tissue in the lymph nodes and distant organs. A recent progress indicated that small-molecule fluorescent imaging was successfully applied clinically to aid glioma surgery (Stummer *et al*, 2006). Therefore, it is feasible to use antibody-conjugated small-molecule fluorophores to identify tumour cells *in vivo.* This technology could aid in several surgical procedures, but more importantly, it could be used to non-invasively characterise biological properties of tumour cells. Currently, small-molecule fluorophores have relatively lower extinction efficiency than QDs, which limits their applications in deep-tissue imaging to detect metastasis. However, this is a very active research area and we anticipate breakthroughs to generate brighter small-molecule fluorophores in the near future to fulfil this gap.

## References

[bib1] Ballou B, Ernst LA, Andreko S, Harper T, Fitzpatrick JA, Waggoner AS, Bruchez MP (2007) Sentinel lymph node imaging using quantum dots in mouse tumor models. Bioconjug Chem 18: 389–396 17263568 10.1021/bc060261j

[bib2] Ballou B, Lagerholm BC, Ernst LA, Bruchez MP, Waggoner AS (2004) Noninvasive imaging of quantum dots in mice. Bioconjug Chem 15: 79–86 14733586 10.1021/bc034153y

[bib3] Berns EM, Klijn JG, van Staveren IL, Portengen H, Foekens JA (1992) Sporadic amplification of the insulin-like growth factor 1 receptor gene in human breast tumors. Cancer Res 52: 1036–1039 1310636

[bib4] Burtrum D, Zhu Z, Lu D, Anderson DM, Prewett M, Pereira DS, Bassi R, Abdullah R, Hooper AT, Koo H, Jimenez X, Johnson D, Apblett R, Kussie P, Bohlen P, Witte L, Hicklin DJ, Ludwig DL (2003) A fully human monoclonal antibody to the insulin-like growth factor I receptor blocks ligand-dependent signaling and inhibits human tumor growth *in vivo*. Cancer Res 63: 8912–8921 14695208

[bib5] Cai W, Shin DW, Chen K, Gheysens O, Cao Q, Wang SX, Gambhir SS, Chen X (2006) Peptide-labeled near-infrared quantum dots for imaging tumor vasculature in living subjects. Nano Lett 6: 669–676 16608262 10.1021/nl052405t

[bib6] Choi HS, Liu W, Misra P, Tanaka E, Zimmer JP, Itty Ipe B, Bawendi MG, Frangioni JV (2007) Renal clearance of quantum dots. Nat Biotechnol 25: 1165–1170 17891134 10.1038/nbt1340 PMC2702539

[bib7] Diagaradjane P, Orenstein-Cardona JM, N EC-C, Deorukhkar A, Shentu S, Kuno N, Schwartz DL, Gelovani JG, Krishnan S (2008) Imaging epidermal growth factor receptor expression *in vivo*: pharmacokinetic and biodistribution characterization of a bioconjugated quantum dot nanoprobe. Clin Cancer Res 14: 731–741 18245533 10.1158/1078-0432.CCR-07-1958

[bib8] Gao XH, Cui YY, Levenson RM, Chung LWK, Nie SM (2004) *In vivo* cancer targeting and imaging with semiconductor quantum dots. Nat Biotechnol 22: 969–976 15258594 10.1038/nbt994

[bib9] Goetsch L, Gonzalez A, Leger O, Beck A, Pauwels PJ, Haeuw JF, Corvaia N (2005) A recombinant humanized anti-insulin-like growth factor receptor type I antibody (h7C10) enhances the antitumor activity of vinorelbine and anti-epidermal growth factor receptor therapy against human cancer xenografts. Int J Cancer 113: 316–328 15386423 10.1002/ijc.20543

[bib10] Hama Y, Koyama Y, Urano Y, Choyke PL, Kobayashi H (2007) Simultaneous two-color spectral fluorescence lymphangiography with near infrared quantum dots to map two lymphatic flows from the breast and the upper extremity. Breast Cancer Res Treat 103: 23–28 17028977 10.1007/s10549-006-9347-0

[bib11] Jackson H, Muhammad O, Daneshvar H, Nelms J, Popescu A, Vogelbaum MA, Bruchez M, Toms SA (2007) Quantum dots are phagocytized by macrophages and colocalize with experimental gliomas. Neurosurgery 60: 524–529; discussion 529–30 17327798 10.1227/01.NEU.0000255334.95532.DD

[bib12] Kim S, Lim YT, Soltesz EG, De Grand AM, Lee J, Nakayama A, Parker JA, Mihaljevic T, Laurence RG, Dor DM, Cohn LH, Bawendi MG, Frangioni JV (2004) Near-infrared fluorescent type II quantum dots for sentinel lymph node mapping. Nat Biotechnol 22: 93–97 14661026 10.1038/nbt920 PMC2346610

[bib13] Knapp DW, Adams LG, Degrand AM, Niles JD, Ramos-Vara JA, Weil AB, O'Donnell MA, Lucroy MD, Frangioni JV (2007) Sentinel lymph node mapping of invasive urinary bladder cancer in animal models using invisible light. Eur Urol 52(6): 1700–1708 17646044 10.1016/j.eururo.2007.07.007 PMC2385787

[bib14] Li SD, Huang L (2008) Pharmacokinetics and biodistribution of nanoparticles. Mol Pharm 5(4): 496–504 18611037 10.1021/mp800049w

[bib15] Linden HM, Stekhova SA, Link JM, Gralow JR, Livingston RB, Ellis GK, Petra PH, Peterson LM, Schubert EK, Dunnwald LK, Krohn KA, Mankoff DA (2006) Quantitative fluoroestradiol positron emission tomography imaging predicts response to endocrine treatment in breast cancer. J Clin Oncol 24: 2793–2799 16682724 10.1200/JCO.2005.04.3810

[bib16] Maloney EK, McLaughlin JL, Dagdigian NE, Garrett LM, Connors KM, Zhou XM, Blattler WA, Chittenden T, Singh R (2003) An anti-insulin-like growth factor I receptor antibody that is a potent inhibitor of cancer cell proliferation. Cancer Res 63: 5073–5083 12941837

[bib17] Nahta R, Esteva FJ (2003) HER-2-targeted therapy: lessons learned and future directions. Clin Cancer Res 9: 5078–5084 14613984

[bib18] Parungo CP, Ohnishi S, Kim SW, Kim S, Laurence RG, Soltesz EG, Chen FY, Colson YL, Cohn LH, Bawendi MG, Frangioni JV (2005) Intraoperative identification of esophageal sentinel lymph nodes with near-infrared fluorescence imaging. J Thorac Cardiovasc Sur 129: 844–850 10.1016/j.jtcvs.2004.08.001 PMC1361260 15821653

[bib19] Perik PJ, Lub-De Hooge MN, Gietema JA, van der Graaf WT, de Korte MA, Jonkman S, Kosterink JG, van Veldhuisen DJ, Sleijfer DT, Jager PL, de Vries EG (2006) Indium-111-labeled trastuzumab scintigraphy in patients with human epidermal growth factor receptor 2-positive metastatic breast cancer. J Clin Oncol 24: 2276–2282 16710024 10.1200/JCO.2005.03.8448

[bib20] Rowinsky EK, Youssoufian H, Tonra JR, Solomon P, Burtrum D, Ludwig DL (2007) IMC-A12, a human IgG1 monoclonal antibody to the insulin-like growth factor I receptor. Clin Cancer Res 13: 5549s–5555s 17875788 10.1158/1078-0432.CCR-07-1109

[bib21] Sachdev D, Singh R, Fujita-Yamaguchi Y, Yee D (2006) Down-regulation of insulin receptor by antibodies against the type I insulin-like growth factor receptor: implications for anti-insulin-like growth factor therapy in breast cancer. Cancer Res 66: 1–12 10.1158/0008-5472.CAN-05-3126 16489046

[bib22] Sachdev D, Yee D (2006) Inhibitors of insulin-like growth factor signaling: a therapeutic approach for breast cancer. J Mammary Gland Biol Neoplasia 11: 27–39 16947084 10.1007/s10911-006-9010-8

[bib23] Simberg D, Duza T, Park JH, Essler M, Pilch J, Zhang L, Derfus AM, Yang M, Hoffman RM, Bhatia S, Sailor MJ, Ruoslahti E (2007) Biomimetic amplification of nanoparticle homing to tumors. Proc Natl Acad Sci USA 104: 932–936 17215365 10.1073/pnas.0610298104 PMC1783417

[bib24] Soltesz EG, Kim S, Kim SW, Laurence RG, De Grand AM, Parungo CP, Cohn LH, Bawendi MG, Frangioni JV (2006) Sentinel lymph node mapping of the gastrointestinal tract by using invisible light. Ann Surg Oncol 13: 386–396 16485157 10.1245/ASO.2006.04.025

[bib25] Soltesz EG, Kim S, Laurence RG, DeGrand AM, Parungo CP, Dor DM, Cohn LH, Bawendi MG, Frangioni JV, Mihaljevic T (2005) Intraoperative sentinel lymph node mapping of the lung using near-infrared fluorescent quantum dots. Ann Thorac Surg 79: 269–277 15620956 10.1016/j.athoracsur.2004.06.055 PMC1421510

[bib26] Steller MA, Zou Z, Schiller JT, Baserga R (1996) Transformation by human papillomavirus 16 E6 and E7: role of the insulin-like growth factor 1 receptor. Cancer Res 56: 5087–5091 8895768

[bib27] Stummer W, Pichlmeier U, Meinel T, Wiestler OD, Zanella F, Reulen HJ (2006) Fluorescence-guided surgery with 5-aminolevulinic acid for resection of malignant glioma: a randomised controlled multicentre phase III trial. Lancet Oncol 7: 392–401 16648043 10.1016/S1470-2045(06)70665-9

[bib28] Tada H, Higuchi H, Wanatabe TM, Ohuchi N (2007) *In vivo* real-time tracking of single quantum dots conjugated with monoclonal anti-HER2 antibody in tumors of mice. Cancer Res 67: 1138–1144 17283148 10.1158/0008-5472.CAN-06-1185

[bib29] Van Rooijen N, Sanders A (1994) Liposome mediated depletion of macrophages: mechanism of action, preparation of liposomes and applications. J Immunol Methods 174: 83–93 8083541 10.1016/0022-1759(94)90012-4

[bib30] Wu JD, Odman A, Higgins LM, Haugk K, Vessella R, Ludwig DL, Plymate SR (2005) *In vivo* effects of the human type I insulin-like growth factor receptor antibody A12 on androgen-dependent and androgen-independent xenograft human prostate tumors. Clin Cancer Res 11: 3065–3074 15837762 10.1158/1078-0432.CCR-04-1586

[bib31] Zhang H, Sachdev D, Wang C, Hubel A, Gaillard-Kelly M, Yee D (2008a) Detection and downregulation of type I IGF receptor expression by antibody-conjugated quantum dots in breast cancer cells. Breast Cancer Res Treat 114(2): 277–285 18418709 10.1007/s10549-008-0014-5

[bib32] Zhang H, Yee D (2004) The therapeutic potential of agents targeting the type I insulin-like growth factor receptor. Expert Opin Investig Drugs 13: 1569–1577 10.1517/13543784.13.12.1569 15566314

[bib33] Zhang H, Yee D (2006) Is the type I insulin-like growth factor receptor a therapeutic target in endometrial cancer? Clin Cancer Res 12: 6323–6325 17085640 10.1158/1078-0432.CCR-06-1707

[bib34] Zhang H, Yee D, Wang C (2008b) Quantum dots for cancer diagnosis and therapy: biological and clinical perspectives. Nanomed 3: 83–91 10.2217/17435889.3.1.83 18393668

